# Illness beliefs about depression among patients seeking depression care and patients seeking cardiac care: an exploratory analysis using a mixed method design

**DOI:** 10.1186/s12888-018-1936-z

**Published:** 2018-11-15

**Authors:** Julia Luise Magaard, Bernd Löwe, Anna Levke Brütt, Sebastian Kohlmann

**Affiliations:** 1 Department of Medical Psychology, University Medical Center Hamburg-Eppendorf, Hamburg, Germany; 2 Department of Psychosomatic Medicine and Psychotherapy, University Medical Center Hamburg-Eppendorf, Hamburg, Germany; 3 Department of Health Services Research, School of Medicine and Health Sciences, Carl von Ossietzky University Oldenburg, Oldenburg, Germany

**Keywords:** Causal beliefs, Coronary heart disease, Depression, Illness representations

## Abstract

**Background:**

Treatment of depression in cardiac patients is difficult. Patients’ illness beliefs regarding depression are associated with outcomes. The aim of the mixed-methods study was to test whether patients in routine care for depression differ from patients with depression in routine care for cardiac diseases regarding illness beliefs about depression.

**Methods:**

A consecutive sample of *n* = 217 patients with depressive disorder was recruited from routine care for depression (*N* = 148) and routine care for cardiac diseases (*N* = 69). Beliefs about depression were measured by the Brief-Illness Perception Questionnaire. Causal beliefs were categorized using qualitative methods. To investigate differences regarding other illness beliefs, we performed an ANCOVA controlling for sociodemographic and clinical differences by propensity score matching.

**Results:**

Patients in routine care for cardiac diseases attributed their depression more often to physical illnesses (48% vs. 16%) and less often to their self (30% vs. 47%), problems at work (25% vs. 35%), childhood (25% vs. 30%), and negative life events (19% vs. 25%) in contrast to patients in routine care for depression. Patients in routine care for cardiac diseases reported beliefs of lower disability, burden, and treatment-control and of higher self-control in contrast to patients in routine care for depression.

**Conclusions:**

Illness beliefs especially causal beliefs differ between patients in routine care for cardiac diseases and routine care for depression. Future research should investigate effects of these illness beliefs. We recommend exploring patients’ illness beliefs about depression in routine care for cardiac diseases and routine care for depression.

## Background

Major depression is one of the most significant clinical disorders with a lifetime prevalence of 11.6% [1]. Depression is among the leading causes of premature death [2], suicide, and the progression of chronic physical conditions such as coronary heart disease [2]. Effective psychotherapeutic and psychopharmacological treatment options are available. In contrast to patients with depression without somatic comorbidity, psychotherapeutic and psychopharmacological treatments have only modest effects on depression severity among patients with depression and cardiac diseases [3–6]. To target and individualize psychological treatments, research is required to investigate the effects of individual factors on treatment [4]. Besides potential biological influences of cardiac risk markers (e.g. thyroid hormones and inflammatory blood markers) on response to depression treatment [7], patients’ illness beliefs have been identified as possible antagonists for effective depression treatment [8, 9]. However, illness beliefs might differ between patients seeking help for depression and depressed patients seeking help for cardiac diseases. Thus, illness beliefs, irrespective of being correct or false, might be dysfunctional and inhibit effective treatment.

Individuals who experience symptoms or are faced with a new diagnosis will develop an organized pattern of beliefs about their health threat including cognitive and emotional representations [10, 11]. In order to build these so-called illness beliefs, individuals use their own knowledge as well as experiences of others with similar symptoms or diagnoses [10, 11]. Leventhal’s common sense model of illness representations states that illness beliefs affect patients’ coping behavior and their appraisal of the outcome of their efforts [11]. According to the model, patients’ illness beliefs are grouped into five dimensions, namely identity, timeline, cause, consequences, and cure/control [10, 11]: The first includes beliefs about the label as well as about symptoms associate with the condition. The second includes beliefs about the duration of an illness ranging from acute to chronic. Causal beliefs are individual conceptions about what had caused an illness. Beliefs about consequences comprise effects of the illness on daily life, whereas cure/control beliefs contain perceived possibilities to cure or to control the condition through treatment or personal behavior [10, 11]. The common sense model is widely used and empirically confirmed among patients with somatic diseases [12, 13] and among patients with depression [14–19]. In patients with heart diseases, it has been shown that illness beliefs about heart failure are associated with psychological well-being [13]. In patients with major depression, illness beliefs affect illness-related behaviors [15, 20] and treatment outcomes like psychological health [8] and quality of life [9]. Illness beliefs may affect depression treatment outcomes in many ways: For example, a considerable number of patients believes that antidepressant use leads to addiction [21], which in turn may explain non-adherent medication intake. A longitudinal study among patients with depression in primary care reported that illness beliefs at baseline influenced the depression severity 6 months later [8]. For instance, beliefs that physical exercise and psychotherapy are helpful to control depression predicted improved depression scores [8]. In terms of help-seeking behavior, a study showed that patients who did not believe in effectiveness of treatment and believed in short-term depression not affecting their everyday lives, did not sought depression treatment [20]. Focusing on patients’ causal beliefs, studies showed that patients’ causal beliefs about depression are associated with severity of depression [22, 23], coping [15, 16], and outcome [18, 23]. Cornwall et al. [22] concluded that biological reasons for patients’ depression are associated with severity of depression. Brown et al. [15] reported that individuals believing stress or interpersonal problems caused their depression, are more likely to vent or blame themselves and exhibit poorer psychosocial functioning. Bann and colleagues [23] have shown that strong beliefs in external causes, i.e. biological abnormality are associated with less improvement. Additionally, the belief in bio-genetic causes is associated with reduced perceived positive outcomes in a sample of patients taking antidepressants [18]. Taken together, the investigation of depression related illness beliefs provides insights how depression treatment can be optimized.

Major depression often occurs comorbid with chronic somatic diseases [24]. Especially in patients with heart diseases, the rate of depression is heightened, and constitutes an independent risk factor for morbidity and mortality [25]. Regarding illness beliefs of depressed patients with comorbid somatic diseases, qualitative studies show that the beliefs regarding different illnesses often interact: Patients experience their conditions as either independent or related in terms of causing each other [26–28], forming far more complex illness representations. A qualitative interview study conducted among primary care patients with depression and a chronic disease (i.e. coronary heart disease or diabetes) showed that they not necessarily considered their depressive symptoms as depression and felt responsible for resisting depression [29]. In line with these findings, patients with depression and chronic heart failure experienced lower levels of cognitive-emotional depression symptoms like depressed mood, worthlessness, or guilt compared to depressed patients without chronic heart failure [30]. Identifying dysfunctional illness beliefs among patients with comorbid heart disease could facilitate an increased awareness of patients’ perspectives and help to establish a more patient-centered care. In addition, understanding these complex depression related illness representations in patients with chronic physical diseases is important, because they appear to impact self-management [27], could have implications for engagement with depression screening [31] and, thus, for the provision of care [27]. With respect to modest effects of depression treatment among patients with heart diseases [3–6], the investigation of depression related illness beliefs appears to be promising.

Taken together, investigating depression related illness beliefs in patients with cardiac disease could unveil new insights into how to optimize care. However, a deeper understanding about how depression related illness beliefs in patients seeking help for cardiac care might differ from patients seeking help for primarily depression care is currently lacking. Accordingly, the aim of this study is to contrast patients with depression in routine care for cardiac disease (RCC) to patients in routine care for depression (RCD) with regard to their depression related illness beliefs.

## Methods

### Study participants and study design

A consecutive sample of *n* = 217 patients was recruited from routine care for depression (*N* = 148) and routine care for cardiac disease (*N* = 69). Patients were included if they had at least moderate depression severity (Patient Health Questionnaire: PHQ-9 ≥ 10) and indicating that they were diagnosed with major depression. Data from patients in RCD is based on a study about help-seeking behavior among patients with depression. Participants in RCD were recruited between August 2015 and May 2016 from three primary care practices (*N* = 25), two psychotherapeutic practices (*N* = 5), a psychiatric outpatient clinic (*N* = 14), and three inpatient clinics (*N* = 104) in Hamburg and in the surrounding area. 218 participants agreed to participate, 156 fulfilled the criteria of PHQ-9 Score ≥ 10 and 8 were excluded because of missing data. Patients in RCC were consecutively recruited between October 2011 and October 2013 from three cardiology centers in Hamburg, Germany. This cross-sectional data is from the DEPSCREEN-INFO trial (ClinicalTrials.gov, Identifier: NCT01879111). DEPSCREEN-INFO is a randomized controlled trial, which examines depression-screening strategies in patients with coronary heart disease or hypertension. Out of 355 cardiac patients with PHQ-9 ≥ 10, 69 patients indicated that they were diagnosed with major depression and filled in a questionnaire about illness beliefs, and thus, were included in the analysis. All patients filled in a questionnaire about patients’ illness beliefs, about sociodemographic characteristics, depression treatment as well as depression severity. Guideline recommended depression treatment was defined as receiving psychotherapy, pharmacotherapy or a combination of both.

### Study variables

Illness beliefs were measured by the Brief-Illness Perception Questionnaire (Brief-IPQ, [32, 33]). Items assess cognitive illness beliefs like consequences, timeline, personal control, treatment control, and identity as well as illness comprehensibility. All of these items are rated using a 0-to-10 response scale. Causal representations are assessed by an open-ended response item, which asks patients to list the three most important causal factors in their illness. Depression severity was measured by the Patient Health Questionnaire-9 (PHQ-9, [34, 35]), ranging from 0 to 27. The following categories regarding severity were used: 10–14 (moderate), 15–19 (moderately severe), and 20–27 (severe) [35]. In addition, age, gender, educational level, and marital status were documented. Among the participants recruited from RCC, severity of cardiac illness was measured by structured self-report measures reflecting the New York Heart Association (NYHA) Functional Classification system as well as the Canadian Cardiovascular Society (CCS) Angina Grading Scale.

### Data analyses

#### Qualitative analyses

A previously developed category system of causal beliefs about mental disorders [36] was used and all statements were deductively assigned to the category system to analyze the qualitative data. The category system describes twelve content-related categories with subcategories, namely “problems at work”, “problems in social environment”, “self/internal states”, “unspecific stress and overload”, “negative life events”, “childhood, youth, parental home”, “physical complaints and illnesses”, “predisposition”, “social situation”, “insufficient treatment”, “fate”, and “lack of causal beliefs” [36]. Three researchers (ALB, SK, JLM) assigned all statements to the category system. An inter-rater reliability of Fleiss Kappa = .76 was accomplished on the level of categories in the categorization process [37, 38], which can be interpreted as a substantial agreement [39]. Mismatching categorizations were discussed until consensus was reached and categorizations were checked to improve coherence. Frequencies of patients stating at least one causal belief referring to a category were calculated. We contrasted these frequencies in patients in RCC to patients in RCD.

#### Quantitative analyses

Before comparing the differences in illness beliefs between the samples, we conducted a propensity score matching (PSM) procedure to minimize the effect of other covariates. Individual propensity scores were calculated through logistic regression modeling based on age, gender, years of formal school education, living situation, and depression severity. PSM was performed according to statistical recommendation [40] using exact matching standard caliper size of 0.2 × log [SD of the propensity score]. Standardized differences were estimated before and after matching to evaluate the balance of covariates. To investigate whether patients in RCD differ from patients in RCC with regard to their illness beliefs, we performed an ANCOVA using the scores of the Brief-IPQ subscales as dependent variable. The PSM score was used as a covariate. Given that multiple tests were performed, a false discovery rate approach (Benjamini-Hochberg procedure) was applied when judging the significance of each test to reduce the risk of alpha inflation [41].

Missing data was less than 2% on every PHQ-9 item and Brief-IPQ item. Thus, missing data were not imputed and all available information was used (pairwise deletion). Analyses were performed using SPSS Version 22.0 (Chicago Inc).

## Results

### Sample description

Sample characteristics are shown in Table 1. On average, participants from RCD had higher educational levels, were younger, to a higher percentage female (73% vs. 55%, *χ*^2^ (2, *N* = 217) = 6.85, *p = .009*)), and unmarried (76% vs. 52%, *χ*^2^ (2, *N* = 217) = 11.99, *p = .001*)) compared to participants from RCC. There was no difference between participants from RCC and RCD with regard to depression severity. On average, both groups were moderately severe depressed (Table 1) and 33% (RCC) vs. 39% (RCD), 45% (RCC) vs. 33% (RCD), and 22% (RCC) vs. 28% (RCD) were classified as moderate, moderately severe, and severe depressed, respectively (*χ*^2^ (2, *N* = 217) = 2.86, *p = .239*)). Using propensity scores as covariates, samples no longer differed regarding age, gender (*χ*^2^ (2, *N* = 69) = 0.26, *p = .614*), formal education, and marital status (*χ*^2^ (2, N = 69) = 2.65, *p = .104*). During study period 48% (*N* = 33) of the patients in RCC and 93% (*N* = 135, N = 2 Missing) of the patients in RCD received guideline recommended depression treatment. Depression severity was positively associated with consequences (*r* = .385, *p* < .001), timeline (*r* = .337, *p* < .001), and identity (*r* = .406, *p* < .001), negatively associated with personal control (*r* = −.213, *p* = .002) and treatment control (*r* = −.156, *p* = .022) and not associated with comprehensibility (*r* = −.058, *p* = .399), irrespective of controlling for RCD and RCC or not.

**Table 1:** Sample characteristics

	Not adjusted			Adjusted		
	RCD (*N* = 148)	RCC (*N* = 69)	*P*-value	RCD (*N* = 148)	RCC (*N* = 69)	*P*-value
Age in years, M (SE)	42.47 (1.06)	59.59 (1.56)	< 0.001	47.88 (0.69)	47.97 (1.10)	.95
≥ 10 years of formal education, M (SE)	0.87 (0.03)	0.55 (0.05)	< 0.001	0.77 (0.03)	0.77 (0.05)	.98
PHQ-9, M (SE)	16.29 (0.34)	16.45 (0.50)	.80	16.34 (0.37)	16.34 (0.59)	.99

Legend: *RCD* routine care for depression, *RCC* routine care for cardiac diseases, *M* mean, *SE* Standard error, *PHQ-9* Patient Health Quesionnaire-9. Dummy coding: years of education, 1 ≥ 10 years of formal education, 0 < 10 years of formal education. Sample characteristics were adjusted using Propensity Score Matching

Among patients in RCC, 59% suffered from coronary heart disease and 41% from hypertension. According to NYHA classification, 20%, 26%, 26%, and 28% of the RCC participants were classified to class I (asymptomatic), class II (mild), class III (moderate), and class IV (severe), respectively. Among patients in RCC, NYHA classification was not significantly associated with depression severity (*r*_*s*_ *(Spearman’s rank correlation)* = 0.126 (*p* = .302)) or with five of the six illness beliefs measured by Brief-IPQ items. An exception is a negative association between the Brief-IPQ item treatment control and NYHA classification (*r*_*s*_ = − 0.271 (*p* = .024)). Regarding angina pectoris, 32% of the participants reported experiencing no symptoms, 39% reported experiencing symptoms without physical activity, 15% reported experiencing symptoms when under light and 15% reported experiencing symptoms when under intense physical activity.

### Causal beliefs of patients in RCD and patients in RCC

**Fig. 1:**
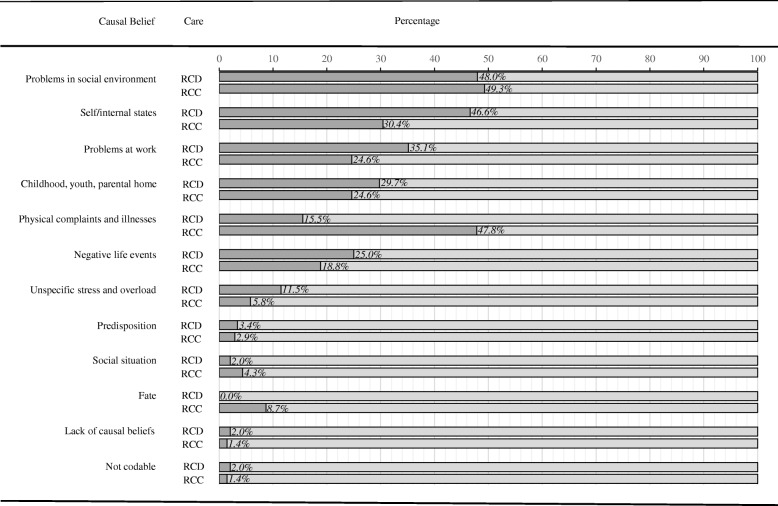
Percentages of patients stating at least one causal belief in that category. Legend: RCD: Routine care for depression (*N* = 148). RCC: Routine care for cardiac disease (*N* = 69). Percentages of patients stated at least one cause referring this category. Insufficient treatment was not included in the bar chart, because it was not mentioned

Figure 1 shows the percentages of patients stating at least one causal belief in a category ordered by frequency in the sample. Stated causal beliefs could be assigned to all causal beliefs categories, apart from insufficient treatment.

Most frequently, stated causal beliefs were assigned to the categories problems in social environment, the self, problems at work, experiences from childhood and youth, physical complaints and illnesses, and negative life events. Patients’ beliefs referring to the category problems in social environment subsumed actual family problems, relationship problems, illnesses of close relatives and their consequences, isolation and lack of appreciation, and private problems. For instance, “drinking problem of my partner” was assigned to illnesses of close relatives. The category self/internal states subsumed statements regarding anxiety, depressive symptoms, and high self-demands whereas the category negative life events consists of statements related to experience of abuse, accidents, and bereavement. Statements about interpersonal problems at work, financial problems as well as problems with working conditions were stated as causal beliefs relating to work. Independently from the care setting, every second patient stated causal beliefs concerning the social environment. Nearly half of the patients with depression in RCC attributed their depression to physical complaints (e.g. pain) and illnesses, whereas only 16% of the patients in RCD stated such causal beliefs. Contrasted to patients in RCC, patients in RCD attributed their depression more often to their self (47% vs. 30%), problems at work (35% vs. 25%), circumstances during childhood and youth (30% vs. 25%), and negative life events (25% vs. 19%).

### Illness beliefs of patients in RCD and patients in RCC

Using propensity score matching (PSM) to adjust for age, gender, years of formal school education, living situation, and depression severity, patients in RCD rated their depression as more disabling in contrast to patients in RCC (Mean (M) Standard error (SE), 7.93(0.19) vs. 6.42(.30), *F* = 15.36, *p* < .001). Patients in RCD had lower beliefs regarding self-control in contrast to patients in RCC (M(SE), 3.93 (0.21) vs. 5.26(.33), *F* = 9.89, *p* = .002). In contrast, patients in RCD had a stronger beliefs that depression treatment would help compared to patients with RCC (M(SE), 6.96(0.22) vs. 5.90(.35), *F* = 5.55, *p* = .02). Patients in RCD also reported higher subjective symptom burden in contrast to patients in RCC (M(SE), 7.49 (0.19) vs. 6.68(.30), *F* = 4.34, *p* = .038). No differences between groups were indicated for timeline and coherence beliefs.

## Discussion

The aim of the study was to contrast patients in RCD to patients with depression in RCC regarding causal beliefs and other illness beliefs using a mixed method approach.

We found that patients with depression in RCC reported physical causal beliefs more frequently in contrast to patients in RCD. Whereas nearly half of the patients in RCC attributed their depression to physical complaints, only 16% of the patients in RCD referred their depression to physical causes. In contrast to patients with depression in RCC, patients in RCD more frequently stated causal beliefs referring to problems at work, self and internal states, circumstances during childhood and youth, and negative life events. Despite the fact that patients were comparable regarding the level of depression severity, patients in RCD differed also from patients in RCC concerning other illness beliefs: Patients in RCD reported higher beliefs about disability, symptom burden, and treatment control compared to patients in RCC. In contrast, patients in RCC reported beliefs of higher self-control compared to patients in RCD.

The patients in both care settings held a variety of different causal beliefs referring to psychosocial problems and physical illnesses predominantly. The content of causal beliefs of patients in the current sample were similar compared to causal beliefs of patients with depression in primary care [42] and in inpatient mental health care [36]. In line with previous qualitative research (e.g. [36, 42]), only a few patients in both settings emphasized the relevance of genetic influences.

Half of the patients in RCC did and half of the patients did not emphasize chronic illness like e.g. coronary heart disease as a cause for their depression. This is in line with mainly qualitative research about causal beliefs among patients with depression and chronic somatic diseases [26, 29], diabetes [27], and heart disease [28]. Studies reported that patients with both conditions experience their illnesses as either related in terms of causing each other or independent [26–28]. Findings from a qualitative study among elderly primary care patients with depressive symptoms and heart disease can provide a deeper insight into patients’ perspective about heart disease causing depression: Patients explained that heart disease can cause depression because of medication side effects, being frightened by the diagnosis, limitations in daily activities through heart disease, and loss of control as a consequence of navigating the health care system [28].

Besides causal beliefs about depression referring to chronic illnesses, our findings indicate that causal beliefs referring to private and work related social problems and internal states are also important among patients with comorbid coronary heart diseases. Similar to patients in RCD, half of the patients in RCC believed in problems in social environment as a major cause of their depression. This is in line with results from SL Alderson, R Foy, L Glidewell and AO House [29], who reported that comorbid primary care patients’ causal beliefs about depression were complex, preferentially referring to external events like bereavement and relationship breakdowns besides chronic illnesses.

Causal beliefs, irrespective of being correct or false, have been associated with impairment [22, 23], coping [15, 16], and outcome [18, 23]. With our design, we were not able to provide evidence for the impact of illness beliefs on coping and outcome. Thus, future research is needed especially among patients with depression in RCD and RCC.

In our study patients in RCC perceived a lower symptom burden, less disability, believed in higher levels of self-control and lower levels of treatment control compared to patients in RCD. These differences cannot be explained by variations between samples regarding depression severity, age, gender, education, and marital status. These differences regarding appraisal and perception of symptoms is in line with results from N Holzapfel, T Müller-Tasch, B Wild, J Jünger, C Zugck, A Remppis, W Herzog and B Löwe [30]. They found that patients with depression and chronic heart failure experience less cognitive-emotional symptoms compared to patients without chronic heart failure. Both groups experience somatic symptoms of depression to a comparable extent [30]. Findings from a systematic review about illness beliefs about depression in primary care patients with chronic physical diseases indicated that some patients believe that depression is a normal part of life [31]. Similarly, a qualitative study about beliefs about depression among GP patients with coronary heart disease or diabetes found out that patients do not necessarily understand their distress as depression, which makes recognition of depression difficult [29]. These findings are in line with our result that patients in RCC believed in lower symptom burden and less disability compared to patients in RCD although both patient groups do not differ with respect to depression severity. In line with our result of higher levels of self-control among patients in RCC, patients with depression and coronary heart disease or diabetes feel responsible to take control of the situation and make the changes needed to work towards cure of depression [29]. Comparing our findings to the results of a study among primary care patients [20], interesting parallels can be discovered between illness beliefs of patients in RCC and GP patients not seeking treatment for depression: GP patients did not belief in effectiveness of treatment and believed in short-term depression not affecting their everyday lives [20]. Compared to patients in RCD, patients in RCC perceived their depression also as less burdening with lower effects on their everyday lives and reported lower beliefs regarding effectiveness of depression treatment. Taken together, in spite of comparable depression severity, illness beliefs among patients in RCC differ from patients in RCD. At the present point of view, we do not know if these illness beliefs among patients in RCC are adaptive (e.g. through less experiences of impairment) or mal-adaptive (e.g. through impeding help-seeking behavior). Further research is needed to investigate if certain illness beliefs impede help-seeking, recognition of depression, and treatment or are even protective regarding subjective impairment. To test whether the different illness beliefs and causal attributions predict depression treatment and health outcomes in RCC, longitudinal study designs are needed.

Our results also provide initial information for tailoring depression treatment regarding the illness beliefs in patients seeking cardiac care. A focus on changing illness beliefs likely to be dysfunctional could help improve depression treatment in cardiac patients. For instance, E Broadbent, CJ Ellis, J Thomas, G Gamble and KJ Petrie [43] demonstrated that an illness perception intervention can change illness beliefs and improve rates of returning to work in myocardial infarction patients. Future research is needed to first identify dysfunctional beliefs and then prove appropriate interventions.

We believe this is the first study to specifically contrast patients in RCC to patients in RCD regarding beliefs about depression. By using a mixed-methods design, results from the qualitative analysis of causal beliefs were enriched by quantitative comparisons of other illness beliefs. Different frequencies of causal beliefs between the samples cannot be attributed to differences regarding depression severity, because both samples do not differ with respect to depression severity. In order to minimize the effects of age, gender, education, marital status and depression severity on the quantitative comparison of illness beliefs between the samples, we conducted a PSM procedure.

As a major limitation of the study, we need to discuss the implications of the differences of the groups besides the health care setting. The fact that patients in the RCC group have cardiac diseases, are older, and thus have a higher chance of having other somatic diseases may have an influence on patients’ causal beliefs in this group. We have no information about prior episodes of depression among both groups and sequence of onset of depression and cardiac disease among the RCC group. We did not assess whether patients seeking depression care were also diagnosed with a somatic disease like cardiac disease, thus, we could not control this factor. In addition, it is possible that patients in the RCD group have sought help for their depression, because they have thought psychosocial reasons could be involved. This is also true for the 35% of RCC patients, currently receiving mental health treatment. Thus, our findings focus on causal beliefs common among patients in a certain health care setting and we cannot draw conclusions about the influence of having or not having cardiac diseases on illness beliefs about depression. Future studies may further investigate whether a diagnosis of a cardiac disease is associated with certain depression-related illness beliefs. Additionally, patients were included based on their self-reported diagnoses of major depression and a depression cut-off, as opposed to having a formal and confirmed diagnosis. Although the PHQ-9 is an established, reliable, and valid screening instrument for screening and severity of depression and a PHQ-9 score of ≥10 has a sensitivity of 88% and a specificity of 88% for major depression [34, 35], it does not replace a thorough evaluation of psychiatric disorders. Including patients who reported the diagnoses of major depression was necessary, as illness beliefs regarding depression can only be asked for, when people are aware of their disease. Nevertheless, this inclusion criterion limits our results to this certain sample. Future research may find ways to assess illness beliefs independently from patients’ awareness of diagnoses. The difference between RCD and RCC sample sizes might be a source of bias. As we analyzed written material about casual beliefs, it was not possible to clarify the statements of participants.

## Conclusions

The results of this study suggest that patients in RCD differ regarding depression related illness beliefs from patients in RCC although patient groups do not differ with respect to depression severity. These results could be used to facilitate an increased awareness of patients’ perspective and to help to establish a more patient-centered care in these settings. Future research among patients with depression and comorbid heart disease should investigate progressions of illness beliefs about depression as well as relationships between illness beliefs about depression and help-seeking and outcomes to develop interventions for patients at risk.

We suggest health service providers examining patients with depression in different care settings to explore the patients’ beliefs about depression in detail. Moreover, mismatching causal beliefs between patient and treatment provider should be uncovered and discussed in order to enhance concordance between patient and treatment provider regarding illness perception and to develop shared treatment plans. Among patients in RCC, psychosocial as well as physical causal beliefs should be considered in order to plan depression interventions.

## Abbreviations

ALB: Anna Levke Brütt
BL: Bernd Löwe
BMBF: German Federal Ministry of Education and Research
Brief-IPQ: Brief Illness Perception Questionnaire
CCS: Canadian Cardiovascular Society
DFG: German Research Foundation
JLM: Julia Luise Magaard
M: Mean
NYHA: New York Heart Association
PHQ-9: Patient Health Questionnaire - 9
PSM: Propensity score matching
RCC: Routine care for cardiac disease
RCD: Routine care for depression
SE: Standard error
SK: Sebastian Kohlmann
